# Increasing Proportion of HIV-Infected Pregnant Zambian Women Attending Antenatal Care Are Already on Antiretroviral Therapy (2010–2015)

**DOI:** 10.3389/fpubh.2019.00155

**Published:** 2019-06-13

**Authors:** Sehlulekile Gumede-Moyo, Jim Todd, Ab Schaap, Paul Mee, Suzanne Filteau

**Affiliations:** ^1^Department of Population Health, Faculty of Epidemiology and Population Health, London School of Hygiene and Tropical Medicine, London, United Kingdom; ^2^School of Public Health, University of Zambia, Lusaka, Zambia; ^3^ZAMBART, School of Public Health, University of Zambia, Lusaka, Zambia

**Keywords:** ANC, PMTCT, ART, pregnant, HIV-infected, coverage

## Abstract

**Introduction:** Accurate estimates of coverage of prevention of mother-to-child (PMTCT) services among HIV-infected pregnant women are vital for monitoring progress toward HIV elimination targets. The achievement of high coverage and uptake of services along the PMTCT cascade is crucial for national and international mother-to child transmission (MTCT) elimination goals. In eastern and southern Africa, MTCT rate fell from 18% of infants born to mothers living with HIV in 2010 to 6% in 2015. This paper describes the degree to which World Health Organization (WHO) guidelines for PMTCT services were implemented in Zambia between 2010 and 2015.

**Method:** The study used routinely collected data from all pregnant women attending antenatal clinics (ANC) in SmartCare health facilities from January 2010 to December 2015. Categorical variables were summarized using proportions while continuous variables were summarized using medians and interquartile ranges.

**Results:** There were 104,155 pregnant women who attended ANC services in SmartCare facilities during the study period. Of these, 9% tested HIV-positive during ANC visits whilst 43% had missing HIV test result records. Almost half (47%) of pregnant women who tested HIV-positive in their ANC visit were recorded in 2010. Among HIV-positive women, there was an increase in those already on ART at first ANC visit from 9% in 2011 to 74% in 2015. The overall mean time lag between starting ANC care and ART initiation was 7 months, over the 6 year period, but there were notable variations between provinces and years.

**Conclusion:** The implementation of the WHO post 2010 PMTCT guidelines has resulted in an increase in the proportion of HIV-infected pregnant women attending ANC who are already on ART. However, the variability in HIV infection rates, missing data, and time to initiation of ART suggests there are some underlying health service or database issues which require attention.

## Introduction

The Joint United Nations Programme on HIV/AIDS super-fast-track framework of ending the AIDS epidemic by 2030 set to reach and sustain 95% of pregnant women living with HIV with lifelong HIV treatment by 2018 globally (1). The achievement of high coverage and uptake of services along the prevention of mother-to-child (PMTCT) cascade is crucial for national and international mother-to-child transmission (MTCT) elimination goals (2). Twenty-two countries in sub-Saharan Africa with a high burden of MTCT were identified as priority countries for intensified support to achieve the UNAIDS HIV elimination goal (2, 3), which included Zambia. A commonly used surrogate marker for programme effectiveness is programme coverage. For PMTCT this would be the proportion of HIV-infected women and exposed infants in a population that access the different components of the PMTCT programmatic cascade (4). Estimates of coverage with PMTCT services among all HIV-infected pregnant women are vital to monitor progress relative to targets, and to secure donor funding for PMTCT programmes (5).

As a result of increased coverage and improved regimens, rates of HIV transmission from mothers to infants during pregnancy and breastfeeding have decreased around the world (6). The largest decline was in eastern and southern Africa, where it fell from 18% of infants born to mothers living with HIV in 2010 to 6% in 2015 (7). In 2017, 210,000 new infections were averted due to PMTCT (8). Some countries in the SSA like South Africa are approaching the very low MTCT rates achieved in high- income countries, but several others such as Zambia, Angola, DRC, Nigeria, Lesotho, and Kenya lag far behind at the moment (9). In Zambia coverage of pregnant women living with HIV accessing antiretroviral medicines was 92% [78–>95] in 2017, a decrease from 95% in 2015 (8).

This paper describes trends in the coverage of PMTCT services from 2010 to 2015 using the SmartCare database of routine clinical information collected in Zambia. This is the first study to have evaluated the effectiveness of implementing post 2010 PMTCT interventions nationwide using SmartCare routine data.

## Methods

### Study Design

This was a retrospective cohort study using routinely collected data. The study population was all pregnant women attending antenatal care (ANC) from January 2010 to December 2015 in health facilities using the SmartCare database.

In Zambia over 90% of pregnant women attend ANC services at least once during their pregnancy, but only 47% deliver at health facilities (10). Thus, it is difficult to ensure that eligible pregnant women receive the complete treatment to prevent transmission of HIV to their babies. Although more than 75% of the ANC facilities currently provide PMTCT services, the majority of these facilities are along the country's main rail line and in urban centers, resulting in geographical inequity (10).

### Data Sources

The study retrospectively analyzed the Ministry of Health electronic SmartCare database, using routinely collected data from all pregnant women attending ANC from January 2010 to December 2015. SmartCare is a Zambian Ministry of Health-led project funded from the United States Centre for Disease Control and Prevention (CDC) (11). The SmartCare database was developed to improve continuity of care and provide timely data on maternal and child health, HIV/AIDS, tuberculosis, and malaria interventions for public health purposes. Since 2005, the SmartCare database has been deployed in over 800 health facilities, which represents 40% of all facilities in Zambia, including the biggest and busiest health facilities. These results come from 886 health facilities from all provinces in Zambia. The Southern province had the most number of facilities (254/886) represented in the dataset, followed by the Copperbelt (187/886), and Eastern (166/886) provinces. Muchinga and Northern provinces had the least number of facilities, 10 and 26, in the analyzed dataset.

### Data Extraction

The data was extracted into Excel, without names, but with the unique identity (ID) number, and then transferred to Stata 13 for cleaning and analysis. All women enrolled in a facility using SmartCare have an electronic health record about their ANC visits which includes information collected in each visit. Records are updated at every point of clinical service. SmartCare is organized into comprehensive modules and sub-modules. The information from various modules is linked through the unique ID number. For this study, the ANC data was linked to the HIV Client Summary module and the ARV Eligibility Interaction Module to identify HIV-positive women. Data from the Obstetric History Module was then used to segregate PMTCT clients from general ART clients. The oldest date of HIV testing and ANC visit date were used to determine whether women had known their HIV status before the ANC visit. The final data were stratified by province using the geography file from the Central Statistical Office (CSO) which has a list of all the districts and provinces.

**Figure 1:**
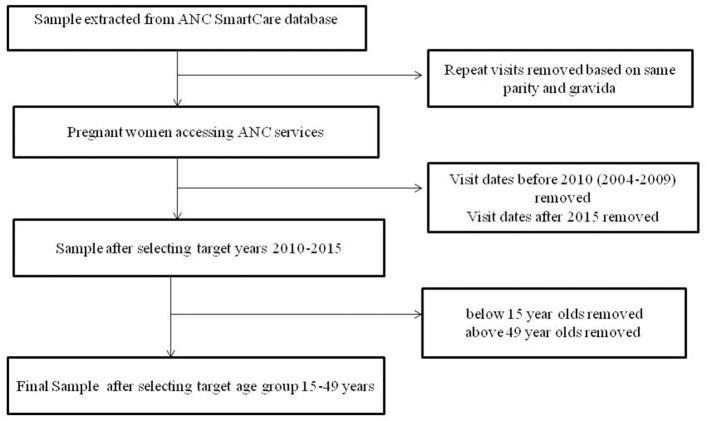
The data flow chart represents the flow chart of participants eligible for inclusion in the analysis.

The first step in data cleaning was to remove duplicate data for repeat visits in the same pregnancy (based on parity and gravid status). This was done by keeping the first visit date of each pregnancy then populating any empty fields with information captured at later visits in the same pregnancy. Records for all the mothers <15 years and those above 49 years of age were dropped from the sample making our target group to be those between 15 and 49 years (reproductive age group). The data flow chart is illustrated in Figure 1. Age groups were categorized as 15–24, 25–34, and 35–49 years. Marital status was grouped into single, married, divorced, widowed, and missing. The education status groups were no education, primary education, junior secondary, secondary, and tertiary education.

### Statistical Analysis

The data were used to estimate the proportion of HIV-positive pregnant women attending ANC by province and year. The study population was divided into three strata: pregnant women with a new HIV test result documented in ANC clinic, pregnant women with known status but not on ART, and pregnant women who were already on ART. Among the total number of pregnant women presenting to ANC clinic in each calendar year; the percentages in each group were calculated.

The STrengthening the Reporting of OBservational studies in Epidemiology (STROBE) guidelines were used to conduct and report on the findings of this study (12).

### Ethics

Ethical approval was granted from Zambia Biomedical Research Ethic Committee (Ref 101-04-16) and the LSHTM Research Ethics Committee (Ref 12086). Permission to use SmartCare data was granted by the Zambia Ministry of Health. The Ethics Committees that approved the study waived the need for written informed consent to be obtained as this was a secondary analysis of previously collected data and the authors had access only to de-identifiable information.

## Results

### Demographics

There were a total of 327,368 visits to antenatal clinics by 172,517 pregnant women in the SmartCare database (Figure 1). However, 64,620 were before 2010, and 2,524 were after 2015. A further 1,052 were under 15 years of age, and 166 were over 49 years of age, leaving104, 155 pregnant women in the final study sample (Table 1) with 33% recorded in 2010. Most women were from Copperbelt (27%), Southern (21%), and Eastern (21%) provinces whilst the fewest where from Luapula and North-western provinces. The majority (51%) were between 15 and 24 years and 82% were married. A high proportion had attained primary level (34%) and secondary level (39%) education, however, educational level attainment data was missing for 20% of pregnant women.

**Table 1:** Demographic characteristics of pregnant women attending ANC in Zambia (2010–2015).

			**Year of Visit**						
			**2010**	**2011**	**2012**	**2013**	**2014**	**2015**	**Total**
Age categories	15–24 years	*N*	16,518	7,124	8,181	8,069	8,224	5,382	53,498
		%	51	51	51	51	53	52	51
	25–34 years	*N*	12,486	5,439	6,162	5,873	5,644	3,881	39,485
		%	38	39	39	37	36	37	38
	35–49 years	*N*	3,494	1,424	1,610	1,750	1,716	1,178	11,172
		%	11	10	10	11	11	11	11
Marital status	Single	*N*	3,125	1,291	1,393	800	783	1,135	8,527
		%	10	9	9	5	5	11	8
	Married	*N*	27,139	11,774	13,124	12,940	12,727	7,569	85,273
		%	84	84	82	82	82	72	82
	Divorced	*N*	149	61	70	52	48	68	448
		%	0.5	0.4	0.4	0.3	0.3	0.7	0.4
	Widowed	*N*	100	69	59	43	52	50	373
		%	0.3	0.5	0.0	0.0	0.0	0.5	0.4
	Missing	*N*	1,985	792	1,307	1,857	1,974	1,619	9,534
		%	6	6	8	12	13	16	9
Education level attained	No education	*N*	1,593	474	1,134	676	657	363	4,897
		%	5	3	7	4	4	3	5
	Primary level	*N*	12,309	4,580	5,631	5,441	4,384	3,026	35,371
		%	38	33	35	35	28	29	34
	Secondary level	*N*	12,770	6,098	6,372	5,291	5,057	4,558	40,146
		%	39	44	40	38	32	44	39
	Tertiary	*N*	646	533	434	434	348	392	2,787
		%	2	4	3	3	2	4	3
	Missing	*N*	5,180	2,302	2,382	3,850	5,138	2,102	20,954
		%	16	16	15	25	33	20	20
Total			32,498	13,987	15,953	15,692	15,584	10,441	104,155

### HIV Test Results

Overall during the study period 9% of pregnant women tested HIV-positive (Table 2) at ANC visits whilst 43% had missing HIV test result records. In addition, 34% of the missing HIV test results were in 2014, whereas only 2% were in 2011. More so, over 60% of HIV test results were missing in Lusaka and Muchinga provinces.

**Table 2:** HIV test result for of pregnant women attending ANC in Zambia (2010–2015).

**Result of HIV test**		**Year of Visit**						
		**2010**	**2011**	**2012**	**2013**	**2014**	**2015**	**Total**
Negative	(*N*)	24,411	10,773	10,209	4,533	348	106	50,380
	(%)	75	77	64	29	2	1	48
Positive	(*N*)	4,375	2,133	1,851	858	29	16	9,262
	(%)	13	15	12	5	0.3	0.2	9
Not Clear	(*N*)	81	16	15	14	0	0	126
	(%)	0.3	0.1	0.1	0.1	0.0	0.0	0.1
Missing	(*N*)	3,631	1,065	3,878	10,287	15,207	10,319	44,387
	(%)	11	8	24	66	98	99	43
Total	*N*	32,498	13,987	15,953	15,692	15,584	10,441	104,155

The overall percentage of HIV-positive pregnant women, who tested for the first time at the ANC decreased from 13% in 2010 to 5% in 2013 and then to 0.15% in 2015. The percentage with missing HIV test results increased from 11% in 2010 to 65% in 2013 and then to 98.8% in 2015 (Table 2).

### ART Initiation

**Figure 2:**
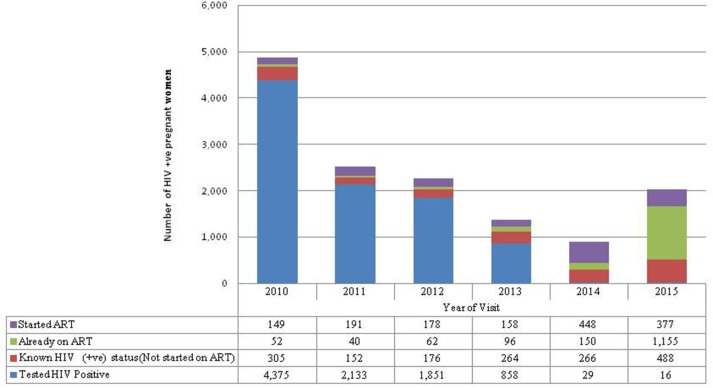
HIV-positive not started on ART refers to women who first tested HIV-positive in this pregnancy but were known HIV not started on ART because they were pregnant when Option A was in place. Started ART refers to women who first tested positive during ANC for this pregnancy and were started on ART. Already on ART refers to pregnant women who were already on ART before seeking ANC services for this pregnancy. Known HIV-positive status refers to women with known HIV-positive status before ANC for this pregnancy so were not tested again but were not started on ART because they were on Option A.

Almost half (47%) of the pregnant women who tested HIV-positive in their ANC visit were recorded in 2010 (Figure 2). More women knew their HIV-positive status in 2015 (30%) than in 2011 (9%). There was a large increase in the proportion of HIV-positive women who were already on ART from 9% of the HIV-positive women seen in 2011 to 74% of the HIV-positive women seen in 2015.

**Figure 3:**
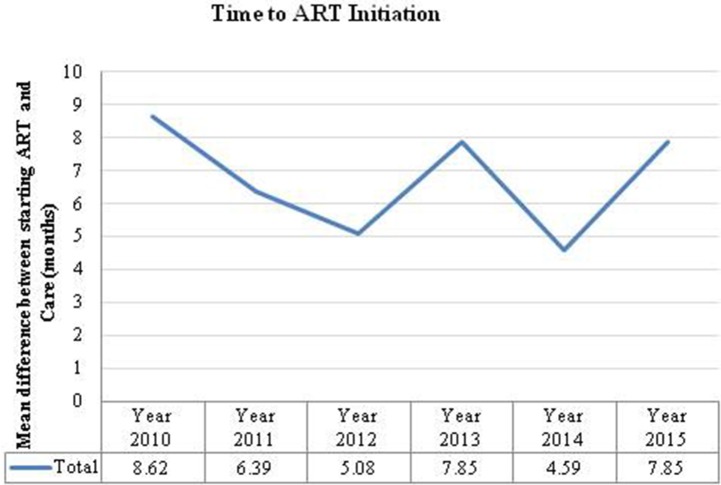
Time to ART initiation is the number of months calculated from the time a women was diagnosed HIV positive to the time they were initiated on ART.

The overall mean time difference between HIV-positive diagnosis at the first ANC visit and ART initiation was 7 months. If a woman was diagnosed at 14 weeks the analysis suggests that most women were not started on ART until after delivery (7–9 months). However, there are notable variations between visit years (Figure 3). There were also large differences between provinces; for example, in 2010 pregnant women in Luapula province took an average of 37 months from diagnosis to treatment whereas in the Copperbelt it took <1 month.

## Discussion

Zambia initiated the PMTC programme in 1999 to address the burden of vertical transmission of HIV and to integrate PMTCT in all maternal, newborn, and child health services throughout the country (13). The results of this study show that, although there is a high rate of engagement with PMTCT services, the variability in HIV infection rates, missing data, and time to initiation of ART suggests there are some underlying health service or database issues which require attention.

Our results indicate a progressive increase in the proportion of women who were already on ART before registering for ANC in their visit year (from 3% in 2010 to 74% in 2015). This is likely to be attributable to the introduction of Option B+ for those women with repeat pregnancies and adoption in 2013 of WHO Test and Treat guidelines that recommend anyone who tests positive for HIV should be started on treatment, regardless of their CD4 count. However, the UNAIDS Prevention Gap Report indicated a decline in pregnant women living with HIV who received effective ART from 96% in 2013 to 87% in 2015 (6). In a systematic review of literature on the effectiveness of implementing post 2010 PMTCT guidelines, we concluded that many HIV-infected women who are engaged in care during pregnancy are lost to follow-up during the postpartum period (14).

The increased volume of patients initiating ART due to test-and-treat and Option B+ could have threatened programme performance and negatively affected the HIV continuum of care for all HIV–infected patients (15). Mathematical modeling using the Lifelong ART tool indicated that the probability of HIV-infected pregnant women initiating ART would increase by 80%. It was also suggested that while the shift would generate higher PMTCT costs, it would be cost-saving in the long term as it spares future treatment costs by preventing infections in infants and partners (16).

Other studies from Africa have shown that the uptake of PMTCT services could be influenced by health system or structural issues such as staffing level, availability, and cost of ART, capacity of health personnel to prescribe appropriate regimens, shortage of supplies in facilities, failure to follow up mothers' or infants' status, and giving wrong information or suboptimal quality of counseling leading to loss or dropout from the PMTCT cascade (17–22). In Zambia lack of human resources remains a serious impediment to addressing HIV, so that even when physical resources are available, there is often not the healthcare personnel to administer them (23). However, knowledge around PMTCT is high: the Zambia Demographic Health Survey (ZDHS) 2013–14, reported that 82% of women and 66% of men were aware of the risk of MTCT and that it can be reduced by taking special drugs during pregnancy (24).

In our study 65% (983/1501) of the women who were initiated on ART after testing HIV-positive during their ANC were documented after the adoption of Option B+ (2013–15). However, increasing ART initiation coverage does not always translate to programme effectiveness: for example, in a surveillance exercise conducted in Lusaka in 2003, 32% of HIV-infected women reported not to actually ingest the NVP tablet given to them in ANC (25). In addition, the risk of being lost to follow up was higher in ‘B+ pregnant' compared to women on ART for their own health in Mozambique (26). In Malawi where Option B+ was first piloted, default, and incomplete adherence were more common with Option B+ than with Option A (27). Hence more efforts must be directed to postnatal programs that ensure retention in care so that women who are initiated on ART do not disengage.

The SmartCare database offers real time data which can enable the Zambian health policy makers to act on urgent PMTCT interventions and improve health care quality and outcomes of mothers and their infants. However, current data are not available for analyses, as there are delays in uploading data to the central database, cleaning, and verifying data, and making the data safe for extraction and this analysis was based on data that were more than 4 years old. SmartCare is a facility-based approach which is unable to account for individuals who do not access ANC services; hence it's possible that we might overestimate PMTCT effectiveness (4). The ANC SmartCare database sample was likely to be biased toward the generally better outcomes of those who receive ANC services. However, with over 90% pregnant women attending ANC services at least once during their pregnancy (24), the results could be a good indicator of program performance.

These results come from more than 800 health facilities from all provinces in Zambia, and hence representative of the population of pregnant women in Zambia, although some provinces, such as Lusaka, may be under represented due to data quality. The level of missing data on HIV test results indicate that this data must be viewed with caution and hence prevents us making meaningful conclusions in the later years (2014–15) where the missing data HIV test results is almost 99%. This was despite the efforts of PEPFAR and the Ministry of Community Development, Mother and Child Health to strengthen the Health Management Information Systems (HMIS) and linkages with the national electronic health record system (28). The main efforts were supposed to be directed toward supporting partners to utilize the capability of SmartCare to electronically populate the HMIS. In contrast, our study found that the SmartCare database has not been mined and data quality has been deteriorating due to the lack of utilization of the data and its findings. We are conducting qualitative research to investigate the problems with using the SmartCare system and how to improve them.

Our study shows that there are major problems in both the completeness of the collection and reporting of data that tracks PMTCT service delivery. The data quality challenges were similar to other studies from the region using routinely collected data (5, 15, 29, 30). Despite tremendous progress and many country-driven successes achieved during the Global Plan, operational challenges in data use, monitoring, and evaluation for PMTCT persist. Collecting longitudinal data on mother–baby pairs throughout the PMTCT cascade is challenging but necessary to optimize maternal and infant outcomes. However, the Global Plan priority countries (which include Zambia) health records are not properly completed, hence the need to scale up electronic data systems (31) such as the SmartCare.

## Limitations

SmartCare data collection is implemented parallel to the main line Ministry of Health HMIS, which also collects HIV test results. This has caused high levels of missing HIV test results data in the SmartCare as clinicians prefer to enter the information in the HMIS forms compared to SmartCare forms which are longer (5–6 pages per interaction). It also means the data take time to be processed and may take 2 years or more before it can be available for analysis.

Due to data security and confidentiality from data custodians, we were not able to get the exact date of birth and the national identity numbers for individuals in the SmartCare database. As a result we could not match records of the same individuals in cases where they are double registered through change of name, or facility. A commonly used surrogate marker for programme effectiveness is programme coverage, i.e., the proportion of HIV-infected/exposed mother/infant pairs in a population that receive a PMTCT intervention (4). In our study the infant-mother pairs could not be linked as infants are registered as separate individuals with unique numbers.

The decrease in the number of records for over 30,000 in 2010 to 10,000 in 2015 is likely to have introduced bias and hence affects the external validity of our study. The findings could have been triangulated against HMIS data as this could provide an opportunity to identify omissions and errors in the dataset.

The data was mainly collected for administrative purposes without research intentions; for example, breastfeeding is part of the PMTCT cascade but the database contains no infant feeding information.

## Conclusion

The implementation of the WHO post 2010 PMTCT guidelines has resulted in an increase in the proportion of HIV-infected pregnant women attending ANC who are already on ART. The SmartCaredatabase could enable Zambian health policy makers to act on urgent PMTCT interventions and improve health care quality and outcomes of mothers and their infants. However, there is a need first to improve procedures for data collection and entry. The missing data observations indicated the need for further qualitative research to determine why it was such a problem.

## Author Contributions

SG-M analyzed the data and wrote the initial draft of the manuscript with guidance from SF and JT. AS, PM, and JT provided advice on cohort datasets and statistical analyses. All authors contributed to subsequent drafts of the manuscript and approved the final version.

### Conflict of Interest Statement

The authors declare that the research was conducted in the absence of any commercial or financial relationships that could be construed as a potential conflict of interest.
